# Application of ChatGPT in Medical Content Development: Challenges and Hope

**DOI:** 10.31661/gmj.v12i.3106

**Published:** 2023-12-01

**Authors:** Akhilesh Vikram Singh, Anudwipa Singh

**Affiliations:** ^1^ Department of Biotechnology, Graphic Era Deemed to be University, Uttarakhand, India; ^2^ Sri Sai Jyothi College of Pharmacy, Hyderabad, Telangana, India

**Keywords:** ChatGPT, Medical Content Development, AI Tool, Clinical Application, Medical Education

## Dear Editor,

Recently, large language models (LLM), specifically “Chat Generative Pre-Trained Transformer” (ChatGPT), has been evidence of artificial intelligence (AI) tool that extensively perform work similar to humans through training on vast amounts of data interlinking with multi-layer recurrent neural networks [1]. As ChatGPT coding language has the potential to produce write-ups like stories and so on, it has capabilities of drafting simulations of scientific abstract writing and medical literature of free grammatical errors, unlike human researchers [2]. ChatGPT has beneficial aspects in several fields, especially academic writing and medical content development. For academic purposes, features of ChatGPT efficiently clear the queries of students; it has a narration of content for providing the prompt and conveyable meaning of text information; through generating codes, it can summarize and provide meaningful essays as well written by students, quality content for research papers, research abstracts created by ChatGPT were indistinguishable by scientists, hence, some writers may seek this tool for a logical application to get massive outputs, unlike traditional laborious manual methods to sort and analyze large volumes of text. It has efficiently pulled the author’s details, research findings, and publication date details. It saves the time of academicians by providing required paper titles and avoids tedious article searches. Also, it is a response giver to the audible conversation to appropriate questions and provides answers; another side, it even rejects incorrect and inappropriate requests [3, 4]. ChatGPT is not a setback in medical writing; it has generated information specific to medical knowledge but also to radiological reports, patient educational information, scientific articles and regulatory documents. It drafts the essential information for clinical trial protocols, study reports and translation of medical information into various languages for comfortable reading and understanding. This has gained significance in diagnosing critical diseases and can write a short case report of a brain tumor l extra-ventricular neurocytoma (EVN), a rare central nervous system tumor. Translational medicine is another medical field that can fill the lag between basic research and clinical practice through the application of ChatGPT by translating the clinical observations of patients to practical amendment, which improves patient medical care for better output. Therefore, AI suits this task well as it can analyze vast data accurately within no time to identify patterns and trends that might not be apparent to humans [5, 6]. ChatGPT-AI technology has made man’s work easy but brought other ethical and unresolved concerns that a human can’t eliminate. However, users face some issues related to violation of the copyright of the content, transparency in AI-generated content used and medico-legal complications. The content credibility, inaccurate results and plagiarism are severe issues found with the medical content developed with the help of LLM tools. The results generated give biased and harmful outputs, leaving us confused about distinguishing between reliable and unreliable sources of information. As a result, the study is left as an irrelevant reference for others [7]. A recent study by Gao et al. evaluated the scientific abstracts written with ChatGPT and original abstracts with plagiarized detector websites and found that original abstracts scored higher with a median ‘plagiarized’ score of 62.5% (IQR 43.25%, 84.75%) compared to with generated abstracts with median ‘plagiarized’ score of 0% (IQR 0, 0)[8]. Similarly, comparing case reports written with ChatGPT and a human author (physician) showed that ChatGPT’s case report lacked the patient’s medical history [9].Sam Altman, a Chief Executive Officer concerned with Open AI’s application, has highlighted that there would be a prominent threat associated with cyber-attacks and disinformation [10]. This would be observed more often at greater significance in the medical field. The training data embedded in AI systems have depicted discriminatory information and the potentiality of the existing stereotypes. By including the generative language models (GLMs) in medical education, a necessary demand is sought to address the existing potential biases. However, several past incidents tagged with Microsoft’s Tay chatbot tweeting racist and sexist content and racial biases in facial recognition technology demonstrate the need for vigilance. Further intellectual property issues, data privacy, and transparency must be eagle-eyed to ensure these tools are used responsibly. Manipulating AI-generated content may mislead medical information or, in some instances, approve illegal or unethical treatments, which would risk the life of medical students and the patient’s critical condition [11]. A two-fold risk is involved when AI-generated information is distributed unauthorizedly, and significant legal and ethical issues may be faced. On the one hand, sharing of AI-simulation models used for medical demonstrations for education purposes and also using patient information without maintaining confidentiality requires necessary copyright permissions, and without getting, it is an illegal issue and mainly causes serious problems as these actions violate privacy laws and copyright regulations; and leaves a potential concern for AI-generated medical content that was unintentionally caused a threat to ethical issues which must be followed during its training phase. These scenarios emphasize keen observation of the data governance protocols and clear usage policies when incorporating AI into medical content development [11]. Further, in the future, the implementation of AI will become essential in medical education and clinical applications, as it will attain a significant hold in clinical support tools for healthcare professionals.

## Conflict of Interest

The authors certify no conflict of interest with any financial organization regarding the material discussed in the manuscript.
